# Fluoride concentration level in rural area in Poldasht city and daily fluoride intake based on drinking water consumption with temperature

**DOI:** 10.1016/j.dib.2017.05.045

**Published:** 2017-06-01

**Authors:** Ali Akbar Mohammadi, Mahmood Yousefi, Amir Hossein Mahvi

**Affiliations:** a *Department of Environmental Health Engineering, Neyshabur University of Medical Sciences, Neyshabur, Iran*; b *Students Research Committee, Department of Environmental Health Engineering, Neyshabur University of Medical Sciences, Neyshabur, Iran*; c *Department of Environmental Health Engineering, Tehran University of Medical Sciences, Tehran, Iran*; d *Center for Solid Waste Research, Institute for Environmental Research, Tehran University of Medical Sciences, Tehran, Iran*

**Keywords:** Drinking water, Fluoride, Daily intake, Poldasht, Iran

## Abstract

Long-term exposure to high level of fluoride can caused several adverse effects on human health including dental and skeletal fluorosis. We investigated all the drinking water source located in rural areas of Poldasht city, west Azerbaijan Province, North West Iran between 2014 and 2015. Fluoride concentration of water samples was measured by SPADNS method. We found that in the villages of Poldasht the average of fluoride concentration in drinking water sources (well, and the river) was in the range mg/l 0.28–10.23. The average daily received per 2 l of drinking water is in the range mg/l 0.7–16.6 per day per person. Drinking water demands cause fluorosis in the villages around the area residents and based on the findings of this study writers are announced suggestions below in order to take care of the health of area residents.

## **Specification Table**

**Table:** 

*Subject area*	*Chemistry*
*More specific subject area*	*Describe narrower subject area*
*Type of data*	*Table and figure*
*How data was acquired*	Spectrophotometer (DR/5000 Spectrophotometer, USA)
*Data format*	*Raw, analyzed,*
*Experimental factors*	*All water samples in polyethylene bottles were stored in a dark place at room temperature until the fluoride analysis.*
*Experimental features*	*Determine the content levels of fluoride*
*Data source location*	*Poldasht, West Azerbaijan province, Iran*
*Data accessibility*	*Data are included in this article and supplement file excel*

## Value of the data

• Drinking water demands cause fluorosis in the villages around the area residents and based on the findings of this study writers are announced suggestions below in order to take care of the health of area residents.
• Ministry of Health notification about the effects of fluoride and food sources with low fluoride.
• Pregnant women prevent from drinking water with high fluoride.
• Ministry of Power settle water in order to reducing fluoride.
• Provision of health water through filtration for drinking and cooking
• Limitation of consumption of some foods including black and white salt, black and white lemon tea, Subpar, and tobacco which have high fluoride content.
• Food interventions such as frequent consumption of fruits and vegetables which contain high rate of antioxidants and milk with high rate of calcium.

## 1 Experimental design, materials and methods

### 1.1 Study area description

**Fig. 1:**
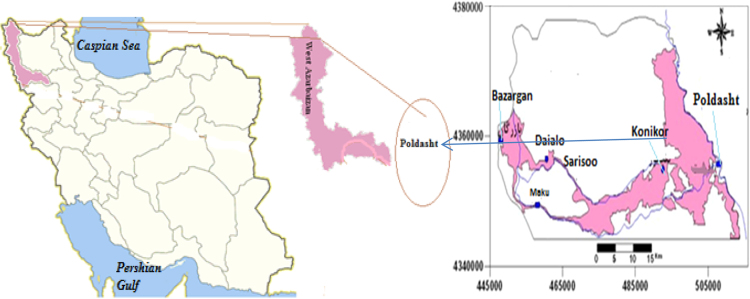
The map and locations of sampling villages.

Poldasht county is located in North West Azerbaijan province of Iran and North Western with coordinates (UTM) *X*=446,625–513,055 to the east and *Y*=4,344,280–4,402,863 is located north latitude. Poldasht meteorological station showed that in a Long-term, the average rainfall was equal to 131.5 mm. The city has also borderline from West and North with Turkey country (Fig. 1).

#### 1.1.1 Determination of the water fluoride concentration

27 drinking water wells in the area were also selected. A total of 128 samples were collected over three consecutive years in 2014–2015. The water samples were collected from the source of drinking water in the sterile plastic 2-l container then transported to the laboratory for water and sewage Poldasht. Fluoride concentration of water samples was determined using SPADNS method according to instruction of Standard. The concentration levels of fluoride in waters were compared with 1053 IR and WHO guidelines for drinking water [1], [2], [3], [4], [5], [6], [7], [8], [9], [10], [11], [12], [13], [14], [15]. Eventually daily fluoride intakes were estimated based on 2 l daily drinking water consumption and concentration levels of fluoride in waters (Table 1).

**Table 1:** Mean concentration levels of fluoride (mg/l) in drinking water of the rural area of Poldasht County of west Azerbaijan province, comparison with EPA and WHO guidelines for drinking water, and daily fluoride intakes with temperature.

*Row*	*Village*	*Source*	*Fluoride concentration* (mg/l)		*Daily intake* (mg/day)	*Average temperature in annual*
			range	mean		
1	pornak	well	2.16–1.57	1.86	3.32	15
2	Moradlo vasati	well	1.46–1.86	1.63	3.26	15
3	Ghoulish lanamish	well	1.59–2.88	2.24	4.48	14
4	Gharghlogh sofla	well	1.54–1.75	1.64	3.28	14.3
5	Nazok sofla	well	0.59–0.63	0.61	1.22	15.1
6	Nazok olyia	well	0.56–0.59	0.575	1.15	15.1
7	Ghir kendi	well	1.53–1.57	1.55	3.1	23
8	divankhane	well+River	0.41–0.61	0.51	1.02	24
9	Shiblo olia	well	0.71–0.81	0.76	1.52	15.1
10	Shahrak aras	Well+River	0.45–0.79	0.62	1.24	15.1
11	Ghore jalo	well	1.66–1.7	1.68	3.36	15.1
12	Eshg abad	well	1.76–1.65	1.7	3.4	15.1
13	Moradlo olia	well	1.92–3	2.46	4.92	15.1
14	sarisoo	well	6.81–8.56	7.63	15.26	15.1
15	Gharghologh sofla	well	1.54–1.86	1.7	3.4	15.1
16	Gharghologh olia	well	1.67–1.75	1.42	2.84	15.1
17	Zakerlo	well	0.28–0.42	0.35	0.7	15.1
18	Shidi	well	0.42–0.86	0.64	1.28	15.1
19	Hasan kandi	Well+ River	0.71–0.8	0.755	1.51	14.3
20	Orooj mohammad	well	1.71–1.84	1.77	3.55	15
21	Bohlol abad	well	2.04–2.55	2.29	4.59	15
22	Ghoch kandi	well	0.78–1	0.89	1.78	15
23	Tape pashi	well	1.5–1.77	1.63	3.27	15
24	Chakhmaghlo sofla	well	0.52–0.56	0.54	1.08	15
25	Eshgh abad	well	1.69–1.66	1.82	3.64	23
26	Daian kendi	well	2.03–2.17	2.1	4.2	15
27	agh otlogh	well	6.3–10.3	8.3	16.6	15
28	1053 IR standard		0.7–1.2			
29	WHO standard		1.5			
